# Co-designing a blueprint for spreading person-centered, Whole Health care to HIV specialty care settings: a mixed methods protocol

**DOI:** 10.1186/s12913-024-11733-2

**Published:** 2024-10-29

**Authors:** Sonia Rupcic, Ming Z. Tam, Kathryn L. DeLaughter, Allen L. Gifford, Anna M. Barker, Barbara G. Bokhour, Chris Xu, Eileen Dryden, Ekaterina Anderson, Guneet K. Jasuja, Jacqueline Boudreau, Jamie H. Douglas, Justeen Hyde, Reagan Mozer, Steven B. Zeliadt, Gemmae M. Fix

**Affiliations:** 1 Center for Health Equity Research and Promotion (CHERP), Veterans Affairs Pittsburgh Healthcare System, Pittsburgh, PA USA; 2 Center for Health Optimization & Implementation Research (CHOIR), Bedford & Boston Veterans Affairs Medical Centers, 200 Springs Rd., Bedford, MA 01730 USA; 3 General Internal Medicine, Chobanian & Avedisian School of Medicine, Boston University, Boston, MA USA; 4 Department of Health Law, Policy, and Management, Boston University School of Public Health, Boston, MA USA; 5 Department of Population and Quantitative Health Sciences, University of Massachusetts Chan Medical School, Worcester, MA USA; 6 Seattle-Denver Center of Innovation (COIN) for Veteran-Centered and Value-Driven Care, Veterans Affairs Puget Sound Health Care System, Seattle, WA USA; 7 Department of Mathematical Sciences, Bentley University, Waltham, MA USA; 8 Department of Health Systems and Population Health, Hans Rosling Center for Population Health, University of Washington School of Public Health, Seattle, WA USA

**Keywords:** Implementation, Spread, Mixed methods, Patient-centered care, Co-design, HIV

## Abstract

**Background:**

Since 2013, the Veterans Health Administration (VHA) has advanced a person-centered, Whole Health (WH) System of Care, a shift from a disease-oriented system to one that prioritizes “what matters most” to patients in their lives. Whole Health is predicated on patient-provider interactions marked by a multi-level understanding of health and trusted relationships that promote well-being. Presently, WH implementation has been focused largely in primary care settings, yet the goal is to effect a system-wide transformation of care so that Veterans receive WH across VHA clinical settings, including specialty care. This sort of system-wide cultural transformation is difficult to implement.

**Methods:**

This three-aim mixed methods study will result in a co-designed implementation blueprint for spreading WH from primary to specialty care settings. Taking HIV specialty care as an illustrative case- because of its diverse models of relationships to primary care - to explore how to spread WH through specialty care settings. We will use the integrated Promoting Action on Research Implementation in Health Services (i-PARIHS) framework to organize quantitative and qualitative data and identify key determinants of WH receipt among Veterans living with HIV. Through a co-design process, we develop an adaptable implementation blueprint that identifies and matches implementation strategies to different HIV specialty care configurations.

**Discussion:**

This study will co-design a flexible implementation blueprint for spreading WH from VHA primary care throughout HIV specialty care settings. This protocol contributes to the science of end-user engagement while also answering calls for greater transparency in how implementation strategies are identified, tailored, and spread.

## Contributions to the Literature

- Using a co-design approach, informed by quantitative and qualitative data, this study will develop an implementation blueprint for spreading Whole Health care (i.e., a person-centered, whole person approach to care, which includes complementary and integrative health services) from primary care into specialty care settings.
- Using data from electronic health records, surveys, qualitative interviews, site visits, and an ongoing evaluation, this study will identify determinants of Whole Health receipt among people living with HIV, receiving care from a national integrated healthcare system.
- This protocol answers calls for more rigorous and systematic approaches to researching and developing system-wide health care transformation while contributing to the science of end-user engagement.

## Introduction

System-wide health care transformations in which new processes are spread across diverse sites are difficult to implement, particularly when they require seismic cultural transformation [1, 2]. For such transformations to be feasible, multiple components need to be adapted to differently situated providers, patients, and clinical contexts. Little is known about how to spread promising interventions across whole systems [3]. “Spreading” refers to the sequential, structured method by which an intervention that is effective in one setting is replicated in another [4]. Due to differences in local contexts, spreading entails some degree of tailoring. Implementation blueprints offer a strategy for tailoring interventions to distinct settings, but proponents of implementation blueprints wonder about their applicability for system-wide transformations [5]. Cara Lewis and colleagues emphasize the role of the blueprint in narrow site-specific contexts but are unsure of the utility of the blueprint for adapting system-wide transformations:

Blueprints could arguably be tailored to the program level, site level, or organization level, depending on interest and resources. Sites within an organization can have significantly different contexts that may lead to unique tailored blueprints; however, it is unclear whether program level tailored blueprints are needed [5]^(p2)^.

Here, Lewis and colleagues question whether the heterogeneity of whole programs, institutions, and systems make them ill-suited to tailoring.

This study is part of a portfolio of work to advance a Whole Health System transformation by developing an implementation blueprint that can be flexibly deployed in different clinical contexts. Since 2013, the Veterans Health Administration (VHA) has advanced a person-centered, Whole Health (WH) System of Care. Whole Health signifies a shift from a disease-oriented system to one that prioritizes “what matters most” to patients in their lives [2]. Whole Health is predicated on patient-provider interactions marked by a multi-level understanding of health and trusted relationships that promote well-being [2]. Current VHA WH implementation efforts are largely focused in primary care settings, yet their goal is to effect a system-wide transformation of care so that Veterans receive care aligned with what matters most, across VHA clinical settings [6, 7]. 

This mixed methods study will develop an implementation blueprint for spreading WH from primary to specialty care settings. The study addresses the concerns Lewis et al. raise by capturing diverse configurations of specialty care in order to develop an adaptable blueprint comprised of both necessary “core” WH elements and “customizable” elements amenable to different types of specialty care settings [8]. We take HIV specialty care settings as a case study for understanding how to spread WH from primary to specialty care settings. Across VHA, HIV specialty care settings represent the diversity in how specialty care is configured in relation to primary care [9, 10]. Subsequent phases of this work will test the fidelity, feasibility, and acceptability of the blueprint developed as part of this study.

Engaging end-users optimizes implementation efforts [11, 12]. Co-design is an increasingly popular methodology that includes end-users (e.g., patients, clinicians, leadership) as partners in the design, development, and implementation of health care interventions [13]. Though co-design methods are insufficiently described and have not been rigorously tested [14, 15], a co-design approach may have advantages for implementation science, as it can help ensure that implementation strategies are applicable and acceptable to end-users in diverse contexts [14]. Our study adopts this approach to account for the diverse configurations of clinical contexts where WH will be spread.

This mixed methods study develops an implementation blueprint capable of supporting the system-wide spread of WH from primary care settings to diverse HIV specialty care settings. Using implementation strategies compiled as part of the Expert Recommendations for Implementing Change (ERIC) [16], we will co-design an implementation blueprint that matches determinants of WH receipt to implementation strategies suited to different HIV specialty care configurations. In doing so, this study contributes to the science of end-user engagement while also answering calls for greater transparency in how implementation strategies are identified, tailored, and spread [5, 14–16]. 

## Methods

### Study Design

Our sequential mixed methods study protocol integrates quantitative (Aim 1) and qualitative (Aim 2) data at the patient, clinic and site level. These findings will then inform a co-design (Aim 3) process with multiple end-users to develop a flexible implementation blueprint for spreading WH care to HIV specialty care settings. We draw from the blueprint methodology described by Lewis and colleagues to aid researchers in tailoring implementation strategies to new settings, service providers, and patient populations [5]. Lewis et al. describe the implementation blueprint as the product of a two-stage process. First, determinants of a practice are identified. Second, strategies are tailored to address relevant determinants across phases of implementation. We will follow this methodology, leveraging co-design to spread WH to specialty care settings.

**Fig. 1:**
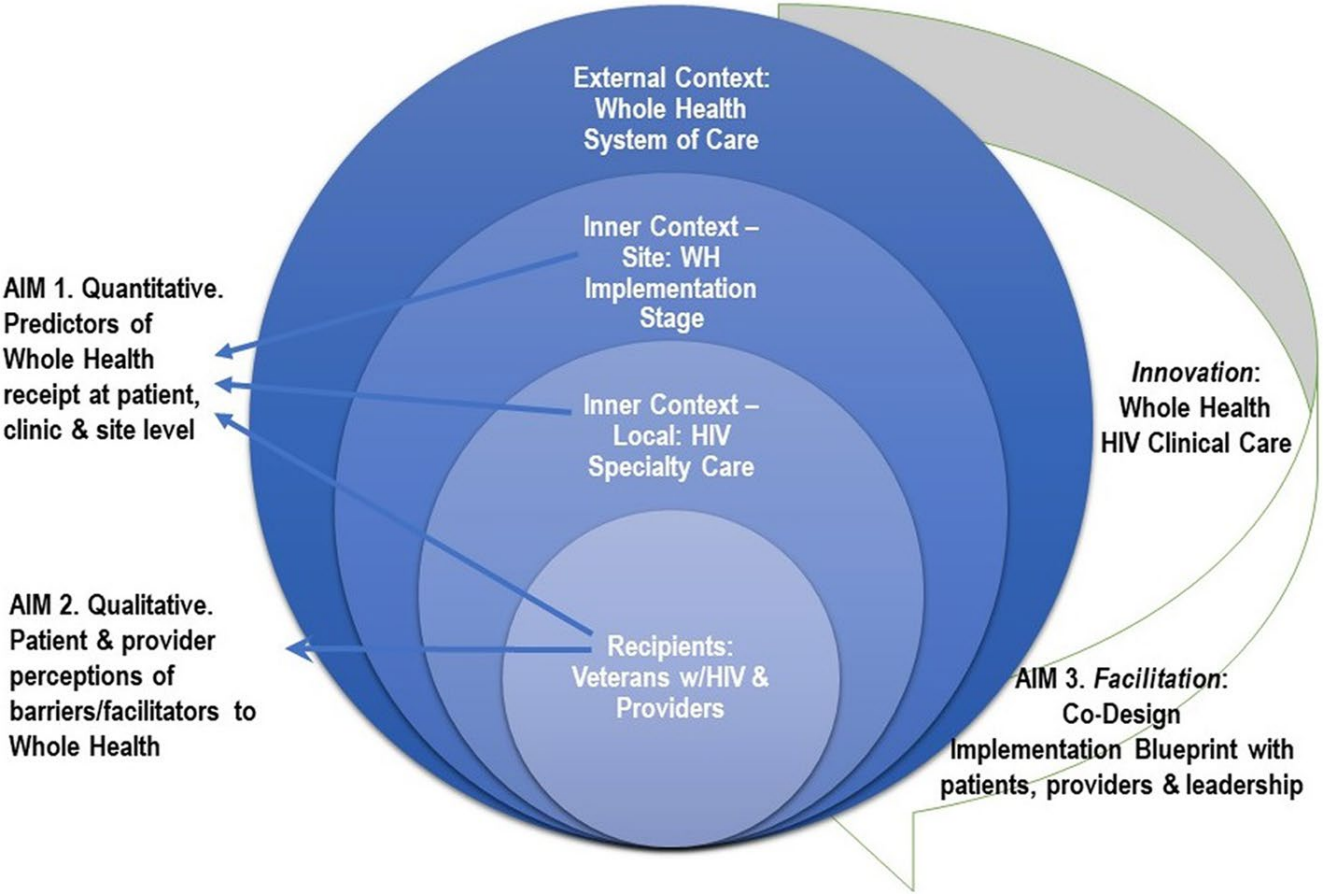
Aims mapped to the i-PARIHS framework

In developing our blueprint, we will be guided by the integrated Promoting Action on Research Implementation in Health Services (i-PARIHS) framework, a conceptual framework that allows us to explicate the dimensions salient to implementation and spread: recipients, innovation, context, facilitation [17]. We will consider end-users throughout this study while incorporating quantitative and qualitative patient, clinic, and site level data (recipients), including data from electronic health records, surveys, operations, interviews, site visits, and co-design consultations to identify determinants of WH (innovation) in diverse HIV specialty care settings (context). The research will culminate in a group of end-users who will aid the study team to interpret findings, identify implementation strategies, and develop an implementation blueprint for spreading WH to diverse specialty care settings (facilitation). See Fig. 1 for an overview of the Aims mapped to i-PARIHS.

### Study Setting

The Department of Veteran Affairs is the largest national integrated health system in the US, with over 150 regional healthcare systems. This study will be based at 18 VA Medical Centers which serve as WH “Flagship” sites, where WH implementation and evaluation efforts have focused [6, 7, 18]. VHA’s WH model is comprised of three components: (1) The Pathway, which provides Veterans with peer support to meet their personal wellness goals; (2) Well-Being Programs, which equip Veterans with self-care skills and may include complementary and integrative health (CIH) (e.g., yoga, tai chi); and (3) WH Clinical Care, where providers and Veterans collaboratively develop a care plan that is meaningfully guided by “what matters most” to Veterans. WH Clinical Care requires a fundamental change in the clinical encounter and not just connecting a Veteran to a peer or service (e.g., health coaching or CIH) [19]. For these reasons, we are especially interested in identifying predictors of receipt of WH Clinical Care, which would be documented as part of a clinical encounter.

We will focus on Veterans living with HIV, their providers, and HIV specialty care settings across the 18 WH flagship sites. The VHA is the US’s largest provider of HIV care, with over 30,000 Veterans living with HIV [20]. HIV specialty care settings have features that parallel many of VHA’s other 17 specialty care service lines. Like other specialty service lines (e.g., endocrinologists treating diabetes), some HIV clinics manage chronic conditions and have an ongoing relationship with their patients [17, 21]. VHA’s HIV specialty settings are notable in that they have a range of models: from serving as a consultative specialty service that coordinates with primary care to functioning as a medical home providing both HIV specialty and primary care consolidated within the HIV clinic [16, 17]. Critically, a WH approach may be particularly valuable for Veterans living with HIV because they are aging, stigmatized, and have a constellation of complex health and social circumstances that will benefit from focusing on “what matters most” [22–26]. We will identify the HIV specialty care models at the 18 WH sites (Aim 1) and use this to purposefully sample for our Aim 2 sites. Our Aim 3 blueprint will then account for these differing specialty/primary care relationships.

### Specific Aims

This mixed methods study has three sequential aims.

- Aim 1: Identify predictors of Whole Health service receipt by Veterans living with HIV.
- Aim 2: Examine Veterans’ and providers’ perspectives on how to spread Whole Health in HIV specialty care settings.
- Aim 3: Engage Veterans living with HIV, providers, and VA leadership to co-design a flexible implementation blueprint that identifies core and customizable components of WH implementation for future spread of WH into HIV specialty care settings.

### Aim 1- Identify Predictors of Whole Health Service Receipt by Veterans Living with HIV

The overarching goal of the first aim is to describe access to WH services by Veterans living with HIV and identify the factors that predict WH receipt. We will identify patient, clinic, and site level predictors of WH services utilization at the 18 Whole Health Flagship sites over a five-year period (2018–2023), a timeframe that coincides with dedicated efforts to support WH implementation. Using VHA electronic health record data, we will create a cohort of Veterans living with HIV using the following criteria: $\:\ge\:$1 inpatient HIV diagnosis; or $\:\ge\:$2 outpatient HIV diagnoses; or $\:\ge\:$1 outpatient HIV diagnosis and $\:\ge\:$2 antiretroviral medications [27]. 

### Dependent Variables

#### Receipt of Whole Health

Using structured data (e.g., procedure and accounting codes) and unstructured data (e.g., clinic notes), we will identify indicators of WH receipt in our cohort during the 2018-23 study period. Our primary outcome will be a dichotomous variable indicating receipt of any WH service. See Table 1 below for definitions. We will also construct two secondary outcomes indicating: (1) the type(s) of WH services received; and (2) the number of WH services received.

**Table 1:** Dependent variable

Outcome	Variables	Source
Veteran Receipt of Whole Health Services	**Whole Health Services** *1. Pathway Services*: WH Pathway, Coaching, WH Education/Skills classes & services. *2. Well-Being Programs*: 2a) Acupuncture, Chiropractic Care, Massage, Yoga, Tai Chi/Qi Gong, Meditation, Guided Imagery, Hypnosis, Biofeedback; 2b) VA community care claims data to identify Acupuncture, Chiropractic Care & Massage services paid for by the VHA but performed by community providers. *3. WH Clinical Care*: Note titles & health factors indicating Personal Health Plan or Personal Health Inventory.	VA administrative data

### Independent Variables

#### Patient-level Variables

To understand how patient level characteristics influence the receipt of WH, we will collect socio-demographic and health data from the VHA Electronic Health Record for the cohort. These data will include race, ethnicity, sex, marital status, age, and rurality (see Table 2). We will describe the overall health and co-morbidities of this cohort using the Elixhauser Comorbidity Index (ECI), a validated summary measure [28]. We will also include chronic pain scores since WH was leveraged as a way to reduce opioid utilization among patients with chronic pain [29–31]. Chronic pain will be defined as a pain score of ≥ 4 (severe pain) where the duration of severe pain lasts more than 30 days.

**Table 2:** Overview of hypotheses, variables & data sources

	Rationale	Hypothesis	Variables	Source
Patient Level	The integration of a WH approach will result in increased use of WH services	Veterans with HIV who are who are rural or older, will be less likely to receive WH services.	• Demographics: Age; Rurality (based on RUCA); Race/ethnicity; Gender; Marital status • HIV status [Combination Antiretroviral Therapy (cART); CD4; HIV Viremia Control, Viral load] • Comorbidities (e.g., chronic pain; diabetes; hypertension; cardiovascular disease; substance or opioid use disorder; mental health) • Social Risk Factors (e.g., employment/financial problems, housing instability, lack of access to transportation, legal issues, nonspecific psychosocial needs, and social/family problems)	VHA Administrative Data
Clinic Level	Understand the setting where Veterans with HIV are receiving WH services	Veterans with HIV who receive care in HIV specialty care clinics providing primary care will be less likely to receive WH services.	• HIV Clinic Model (Consolidated HIV & primary care clinic vs. HIV specialty care) • HIV Clinic Providers’ Whole Health Exposure	Survey; VHA Employee Education Data
Site Level	Examine whether stage of WH transformation predicts receipt of WH	Veterans who receive care at sites lowest in WH implementation stage (“Preparation” or “Foundational” stages) will be less likely to receive WH services.	Whole Health Implementation Stage • Designation Model Self-Assessment Preparation; Foundational; Developmental; Full)	VHA Operations Data

HIV-related health measures will include CD4 cell count and viral load. We will use the relevant Logical Observation Identifiers, Names and Codes (LOINC) laboratory codes to capture CD4 counts for each patient. Viral loads are defined as undetectable (< 20 copies/ml), unsuppressed (20 copies/ml), or missing.

In a way that decenters genetic predispositions and behavioral models of health, WH emphasizes “upstream” determinants of health and well-being [2]. Using a combination of health factors, stop codes, and ICD-10-codes [32], we will characterize the cohort in terms of social risk factors, including employment/financial problems, housing instability, lack of access to transportation, legal issues, nonspecific psychosocial needs, and social/family problems. All patient level variables will be measured at the earliest encounter on or after each patient’s first primary care visit during the study period, which is the earliest point at which we expect that patients will receive WH services. Patient level variables that predict receipt of WH will be used to sample Veteran participants for Aim 2 interviews.

#### Clinic-level Variables

Because WH has predominantly been implemented in primary care settings, the relationship between HIV specialty care and primary care may be a determining factor in whether a Veteran receives WH. In settings where care is consolidated in a standalone HIV clinic, Veterans living with HIV may be less likely to receive WH than Veterans who receive a substantial portion of their care in primary care settings and only HIV-specific care from HIV specialists. HIV clinic level data will be gathered using a combination of survey and employee education data to understand which HIV clinics operate like primary care settings and the extent to which clinic staff have been exposed to WH. Surveys will be administered to Lead HIV Clinicians at the 18 sites.

#### Site-level Variables

Across the 18 WH Flagship sites, WH has varied in implementation. WH receipt may be determined by how well a site implements WH. To understand WH service delivery at each VA medical center, we will use site level data about WH implementation collected and maintained by VA as part of an ongoing program evaluation [7]. The WH System Self-Assessment Tool assigns an ordinal score to each VHA site, quantifying the level of WH implementation (preparation, foundational, developmental, and full) across seven domains [18]. Site-level information will be used in our inferential model and to determine which sites will be included in Aim 2, qualitative data collection.

#### Aim 1. Statistical Analysis

Descriptive statistics will be examined across all dependent and independent variables for our sample. It will include means, standard deviations, and 95% confidence intervals for continuous variables and proportions, odds ratios, and 95% confidence intervals for categorical variables. Our inferential model is described below.

Using patient, clinic, and site level variables as predictors, we will fit a mixed effects binomial logistic regression for the primary outcome (receipt of WH services). We will follow a similar approach in our analyses of the secondary outcomes, using a mixed effects multinomial logistic regression to model the type(s) of WH services received, and a mixed effects Poisson regression to model the number of WH services received. Each model will be fit using random effects at both the clinic and site levels to account for clustering (i.e., patients nested within clinics and sites). Predictors identified during Aim 1 will inform lines of inquiry in Aim 2 and will be matched with implementation strategies to address patient, clinic, and site-specific determinants of WH implementation as part of Aim 3.

We hypothesize that Veterans with HIV will be less likely to receive Whole Health services if they: (1) are rural and/or older (because they are less likely to be offered WH); (2) receive care from an HIV service that functions as a consolidated HIV-primary care clinic; (3) receive care at sites lowest in Whole Health implementation.

#### Aim 1. Power Analysis

A priori sample size calculations were conducted through a multilevel simulation analysis [33, 34]. We consider power for the effect of a single binary variable in a three-level mixed-effects logistic regression model at the two-tailed 0.05 significance level. As effect size estimates from previous work are not available, we calculate the sample size required to detect an odds ratio of 1.5, considered a “small” standardized effect size based on conventional guidelines [28]. Assuming a baseline probability of WH use for all patients of 6%, we will need a total sample size of at least 3,100 patients with HIV, or approximately 172 patients at each of the 18 sites, to detect an effect with nominal (80%) power. Based on our anticipated sample sizes at each site, we will have sufficient power to identify significant predictors of WH use with even small effect sizes.

### Aim 2-Examine Veterans’ and Providers’ Perspectives on how to Spread Whole Health in HIV Specialty Care Settings

Aim 2 will use site visits and semi-structured, qualitative interviews to explore Veterans’ and providers’ perspectives on spreading WH in HIV specialty care. The goal is to understand patient and provider level barriers and facilitators to WH Clinical Care receipt for Veterans living with HIV. For all participants, we will document verbal informed consent.

### Sampling and Setting

Aim 2 will use maximum diversity sampling to ensure the Aim 3 blueprint accounts for variation in patient-level characteristics; clinic-level variations in the model of HIV care (HIV as specialty care service vs. consolidated HIV and primary care in the HIV clinic); and site-level differences in level of WH implementation [35]. Based on Aim 1 findings, will identify 4 sites differing on two parameters: (1) configurations of clinic-level HIV care and (2) site-level WH implementation (see Table 3). Along the dimension of HIV-primary care consolidation, 2 of the sites will have consolidated HIV-primary care clinics while 2 sites will have HIV specialty care clinics focused on HIV, that serve a more limited consultancy role to primary care. Along the dimension of WH implementation, 2 of the WH Flagship sites will have high levels of WH implementation (developmental/full); 2 will have low levels of WH implementation (preparation/foundational).

**Table 3:** Sampling Framework based on HIV Specialty Care Model & Whole Health Designation

	Lower Whole Health implementation Stage (preparation or foundational)	Higher Whole Health implementation Stage (developmental or full)
*HIV Clinic Model*: Consolidated HIV + primary care in the HIV clinic	SITE 1 Patient interviews: up to 15 Provider interviews: up to 10	SITE 2 Patient interviews: up to 15 Provider interviews: up to 10
*HIV Clinic Model*: HIV specialty care serves in a consultancy role to primary care	SITE 3 Patient interviews: up to 15 Provider interviews: up to 15	SITE 4 Patient interviews: up to 15 Provider interviews: up to 15

We will recruit up to 15 Veterans and 10–15 providers at each site (see Table 3) for a total sample of up to 60 Veterans and 50 providers. For sites with a consolidate HIV care model, we will interview up to 10 providers; for sites where patients receive both HIV specialty and primary care, we will interview up to 15 providers to account for the larger pool of providers responsible for care of Veterans living with HIV. Using our Aim 1 cohort, we will recruit Veterans who have received care from one of the four selected sites during the last two years. We will purposively recruit a demographically diverse (race, gender, age, rurality, comorbidity) group with a mixture of WH receipt (e.g., none, WH Clinical Care). Providers from the four sites may include physicians, nurses, nurse practitioners, physician assistants, social workers, or psychologists. At the sites where Veterans with HIV receive care from both HIV and primary care, we will also recruit primary care providers.

#### Aim 2. Data Collection and Analysis

We will conduct site visits at each of the four HIV clinics to understand in-depth these different configurations of HIV and primary care, and the current and potential role of WH. Each site visit will be done by two qualitative methods experts. They will conduct observations and qualitative interviews clinic. Observations will capture clinic composition, workflow, and the site’s WH practices using a semi-structured observation template [36]. 

Our semi-structured qualitative interview guide will adapt constructs from the i-PARIHS framework. To elucidate the i-PARHIS construct, Veterans (recipients) will be asked about where they receive HIV care (context); their experiences with person-centered and WH care (innovation); predictors identified as part of Aim 1 (facilitation); and their preferences on how WH could fit into their current HIV care, with attention to coordination with primary care (innovation; facilitation). During provider interviews (recipients), we will explore their role in caring for Veterans living with HIV (context) and how WH does or could play a role in care (innovation).

To develop insights into how WH might be implemented in HIV care, we will use vignettes describing typical patient scenarios in which providers might use a WH approach. These vignettes were developed and tested as part of earlier pilot research [32]. Interviewers will also ask providers to reflect on Aim 1 determinants of WH receipt and elicit information about how the clinic-level organization of HIV care prevents or enables utilization of WH services. At the end of both the patient and provider interviews, interviewers will request participants’ permission for possibly being recontacted to join the Aim 3 co-design team.

The team will conduct observations during site visits, recording descriptive and analytic fieldnotes as part of data collection. These will be combined with the transcripts for analysis. We will analyze Aim 2 data using deductive and inductive thematic coding. We will code for WH-relevant constructs identified a priori and developed through prior research [10, 37, 38] as well as determinants identified in Aim 1. As we inductively identify new constructs, and add them to the codebook. The team will analyze transcripts as they become available, meeting regularly to establish consensus and iteratively build the codebook. Once coding has been completed, we will organize data into a matrix, identifying themes within the same constructs and across participants. Once the matrix analysis is complete, we will develop a set of four illustrative scenarios representing varying configurations of WH stage and HIV care model (see Table 3) where WH might be spread. Each scenario will outline the determinants to WH receipt identified through Aims 1 and 2, with determinants that are common to all four scenarios noted and compiled into a list.

### Aim 3-Engage Veterans Living with HIV, Providers, and VA Leadership to Co-design a Flexible Implementation Blueprint that identifies Core and Customizable Components of WH Implementation for Future Spread of Whole Health into HIV Specialty Care Settings

Working with key end-users (Veterans living with HIV, providers, and both local and national leadership), we will iteratively co-design implementation strategies for spreading WH into HIV specialty care settings. Aim 3 supports the facilitation element of i-PARIHS, by engaging recipients in interactive problem-solving to co-create implementation strategies that can be tailored to differing contexts (i.e., different HIV Clinic models and sites’ WH stage).

Building on Aims 1 and 2 findings, we will co-design an implementation blueprint for spreading WH into diverse HIV specialty care contexts. Our work will be guided by the four illustrative scenarios of different types of configurations of clinic-level HIV care organization and site-level WH implementation findings developed at the end of Aim 2. Aim 1 and 2 data will be presented in aggregate to facilitate discussion. As such, we will seek approval from the institutional review board for this advisory aim to be designated non-research [39]. 

#### Co-Design

Information about the models of HIV specialty care will guide the identification of core and customizable components of a blueprint with flexible implementation strategies responsive to the needs and experiences of end-users and local context [8]. 

The co-design team will be comprised of researchers, Veterans (Aim 2), providers (Aim 2) and VA leadership. It will include six Veterans living with HIV (sampled to capture a variety of sites, demographics, and life experience), six providers (two HIV providers, two HIV leads, a primary care provider, and a local leader); and VHA leadership directly involved in championing and scaling WH. To build the team, we will have one-on-one virtual discussions with prospective team members, to discuss co-design procedures, expectations, and blueprint goals and build rapport.

The research team will hold virtual co-design sessions to discuss the current state of WH implementation in HIV specialty care, using the Aim 2 scenarios. We will invite co-designers to visualize a desired future, ideal state. In these sessions, we will engage the team in discussions of implementation strategies outlined in ERIC and how they might be matched with determinants described for each scenario [16]. After the introductory sessions, Veterans, providers and leadership will iteratively join the research team in co-design sessions that focus first on the current state of WH and HIV care and then an ideal future state for spreading WH into HIV care. Attendees will participate in whiteboard activities and group discussion. We will use the scenarios to walk co-designers through the clinical appointments, to further visualize areas for potential change. These discussions will help us prioritize implementation strategies.

Guided by a synthesis of Aims 1 and 2 data, the goal is to elicit from the team potential core components of an implementation blueprint that align with determinants that are common across the four scenarios and customizable components that match determinants that are particular to specific scenarios. These components will be discussed and prioritized. Next, we will summarize feedback, compiling a blueprint with core and customizable components alongside the scenarios for their implementation.

#### Implementation Blueprint

Aim 3 will be guided by our goal to leverage end-user expertise and iteratively incorporate their perspectives to develop a suite of implementation strategies which map to ERIC. The resulting blueprint will include key implementation features (e.g., the aim and purpose of the implementation; scope of the change; timeframe and milestones; performance and progress measures) and specify the actor, the action, action targets, temporality, dose, implementation outcomes [5, 16]. As a final step, we will share the finalized blueprint with VHA leadership. In future phases of our research, we will conduct trials to determine the reach, effectiveness, adoption, implementation, and maintenance [40]. 

## Discussion

Research into how to tailor implementation to local contexts is in its early stages. Even less is known about how to spread interventions institutionally in order to effect system-wide transformation. Using HIV specialty care as a case, we will adapt the blueprint methodology proposed by Lewis and colleagues in this journal to co-design a flexible implementation blueprint for spreading WH into HIV specialty care. Findings from this study will facilitate an implementation approach grounded in real-word experience and real-world data, that will enable greater uptake of WH in specialty care settings. Although the conceptual and philosophical cases for whole health and for co-design are compelling and widely endorsed [14], there is almost no evidence about the best practices for rolling out whole health models of care in healthcare systems [13]. Developing such evidence requires clarity and transparency in the protocols being tested, the intervention adaptation strategies planned [41], and measures that will be used to evaluate success. This protocol paper answers an urgent call by implementation researchers for greater transparency in explaining both co-design and implementation blueprint methodologies [14, 15, 41]. 

### Anticipated Challenges and Limitations

The study hinges on the participation of interested parties in interviews and co-design. It is possible HIV specialty providers will not be interested in spreading WH to HIV specialty care. However, this eventuality seems unlikely as findings from our pilot work suggest that most HIV providers are not just amenable but enthusiastic about this person-centered form of care [32]. Many HIV providers see the value of such an intervention for their patient population, noting that the heavy burden of social risk factors in this population [23, 42] means they have much to benefit from the holistic, “all-hands-on-deck” approach implied by WH.

VHA has a robust system for engaging Veterans in implementation research and development [43]. Veterans participate extensively in VHA research [39, 44], including research on HIV interventions [38]. Administrative data imperfectly capture exposure to the WH approach to care. As such, our measurement of WH utilization may over- or underestimate WH service utilization. Finally, Whole Health is being implemented across VHA. Blueprints developed for adapting Whole Health to VHA specialty care settings may have limited generalizability to other healthcare systems. Nevertheless, VHA is the largest national, integrated health care system in the United States and a leader in person-centered approaches. By including diverse HIV specialty care settings in our study, we aim to increase the purchase of our results beyond VHA.

## Data Availability

No datasets were generated or analysed during the protocol development.

## References

[1] Best A, Greenhalgh T, Lewis S, Saul JE, Carroll S, Bitz J. Large-System Transformation in Health Care: a Realist Review. Milbank Q. 2012;90(3):421–56. 10.1111/j.1468-0009.2012.00670.x. 22985277 10.1111/j.1468-0009.2012.00670.x PMC3479379

[2] Krist AH, South-Paul J, Meisnere M, editors. Achieving whole health: a New Approach for veterans and the Nation. National Academies; 2023. 10.17226/26854. 37184190

[3] Charns MP, Lerner B, Yakovchenko V, et al. Achieving transformation to lean management systems in health care. Health Serv Res. 2023;58(2):343–55. 10.1111/1475-6773.14072. 36129687 10.1111/1475-6773.14072 PMC10012231

[4] Greenhalgh T, Papoutsi C. Spreading and scaling up innovation and improvement. BMJ. 2019;365:l2068. 10.1136/bmj.l2068. 31076440 10.1136/bmj.l2068 PMC6519511

[5] Lewis CC, Scott K, Marriott BR. A methodology for generating a tailored implementation blueprint: an exemplar from a youth residential setting. Implement Sci. 2018;13(1):68. 10.1186/s13012-018-0761-6. 29769096 10.1186/s13012-018-0761-6 PMC5956960

[6] Kligler B, Hyde J, Gantt C, Bokhour B. The Whole Health Transformation at the Veterans Health Administration: moving from what’s the Matter with you? To what matters to you? Med Care. 2022;60(5):387. 10.1097/MLR.0000000000001706. 35283434 10.1097/MLR.0000000000001706

[7] Bokhour BG, Hyde J, Zeliadt S, Mohr DC. Whole health system of care evaluation—a progress report on outcomes of the WHS Pilot at 18 Flagship Sites. Httpswww Va GovWHOLEHEALTHprofessional-Resour-ToolsEvidence-Based-Res Asp2020. Published online 2020.

[8] National Academies of Sciences E, Education D, of B and SS and, Board on Children Y, Committee on Fostering Healthy Mental E. Effective Implementation: Core Components, Adaptation, and Strategies. In: Fostering Healthy Mental, Emotional, and Behavioral Development in Children and Youth: A National Agenda. National Academies Press (US); 2019. Accessed 9 July 2024. https://www.ncbi.nlm.nih.gov/books/NBK551850/. 31869055

[9] Bokhour BG, Bolton RE, Asch SM, et al. How should we organize care for patients with human immunodeficiency virus and comorbidities? A Multisite qualitative study of Human Immunodeficiency Virus Care in the United States Department of Veterans affairs. Med Care. 2021;59(8):727. 10.1097/MLR.0000000000001563. 33900271 10.1097/MLR.0000000000001563

[10] Fix GM, Asch SM, Saifu HN, Fletcher MD, Gifford AL, Bokhour BG. Delivering PACT-Principled care: are Specialty Care patients being left behind? J Gen Intern Med. 2014;29(2):695–702. 10.1007/s11606-013-2677-9. 10.1007/s11606-013-2677-9 PMC4070239 24715390

[11] Colquhoun HL, Squires JE, Kolehmainen N, Fraser C, Grimshaw JM. Methods for designing interventions to change healthcare professionals’ behaviour: a systematic review. Implement Sci. 2017;12(1):30. 10.1186/s13012-017-0560-5. 28259168 10.1186/s13012-017-0560-5 PMC5336662

[12] Elwy AR, Maguire EM, Kim B, West GS. Involving Stakeholders as Communication Partners in Research Dissemination efforts. J Gen Intern Med. 2022;37(1):123–7. 10.1007/s11606-021-07127-3. 35349022 10.1007/s11606-021-07127-3 PMC8993948

[13] Bombard Y, Baker GR, Orlando E, et al. Engaging patients to improve quality of care: a systematic review. Implement Sci. 2018;13(1):98. 10.1186/s13012-018-0784-z. 30045735 10.1186/s13012-018-0784-z PMC6060529

[14] Slattery P, Saeri AK, Bragge P. Research co-design in health: a rapid overview of reviews. Health Res Policy Syst. 2020;18(1):17. 10.1186/s12961-020-0528-9. 32046728 10.1186/s12961-020-0528-9 PMC7014755

[15] Chambers DA, Emmons KM. Navigating the field of implementation science towards maturity: challenges and opportunities. Implement Sci. 2024;19(1):26. 10.1186/s13012-024-01352-0. 38481286 10.1186/s13012-024-01352-0 PMC10936041

[16] Powell BJ, Waltz TJ, Chinman MJ, et al. A refined compilation of implementation strategies: results from the Expert recommendations for Implementing Change (ERIC) project. Implement Sci. 2015;10(1):21. 10.1186/s13012-015-0209-1. 25889199 10.1186/s13012-015-0209-1 PMC4328074

[17] Harvey G, Kitson A. PARIHS revisited: from heuristic to integrated framework for the successful implementation of knowledge into practice. Implement Sci IS. 2016;11:33. 10.1186/s13012-016-0398-2. 27013464 10.1186/s13012-016-0398-2 PMC4807546

[18] OPCCCT. Designation Framework for Whole Health implementation: an Organizational Guide to Whole Health Transformation and implementation of the VA Whole Health System. Office of Patient-Centered Care and Cultural Transformation, U.S. Department of Veterans Affairs; 2022.

[19] Anderson E, Wiener RS, Molloy-Paolillo B, et al. Using a person-centered approach in clinical care for patients with complex chronic conditions: perspectives from healthcare professionals caring for veterans with COPD in the U.S. Veterans Health Administration’s Whole Health System of Care. PLoS ONE. 2023;18(6):e0286326. 10.1371/journal.pone.0286326. 37352241 10.1371/journal.pone.0286326 PMC10289382

[20] Maguire E. Caring for Veterans with HIV. HIV.gov. Published 2018. Accessed 9 July 2024. https://www.hiv.gov/blog/caring-veterans-hiv.

[21] Jasuja GK, Reisman JI, Rao SR, et al. Social Stressors and Health among older transgender and gender diverse veterans. LGBT Health. 2023;10(2):148–57. 10.1089/lgbt.2022.0012. 36454239 10.1089/lgbt.2022.0012 PMC10081710

[22] Aidala AA, Wilson MG, Shubert V, et al. Housing Status, Medical Care, and Health outcomes among people living with HIV/AIDS: a systematic review. Am J Public Health. 2016;106(1):e1–23. 10.2105/AJPH.2015.302905. 26562123 10.2105/AJPH.2015.302905 PMC4695926

[23] Menza TW, Hixson LK, Lipira L, Drach L. Social determinants of Health and Care outcomes among people with HIV in the United States. Open Forum Infect Dis. 2021;8(7)ofab330. 10.1093/ofid/ofab330. 34307729 10.1093/ofid/ofab330 PMC8297699

[24] Fix GM, Hyde JK, Bolton RE, et al. The moral discourse of HIV providers within their organizational context: an ethnographic case study. Patient Educ Couns. 2018;101(12):2226–32. 10.1016/j.pec.2018.08.018. 30131263 10.1016/j.pec.2018.08.018 PMC7819576

[25] Wang EA, McGinnis KA, Long JB, et al. Incarceration and Health outcomes in HIV-Infected patients: the impact of Substance Use, Primary Care Engagement, and antiretroviral adherence. Am J Addict Am Acad Psychiatr Alcohol Addict. 2015;24(2):178–84. 10.1111/ajad.12177. 10.1111/ajad.12177 PMC4397180 25662297

[26] Wang EA, McGinnis KA, Goulet J, et al. Food Insecurity and Health: data from the veterans Aging Cohort Study. Public Health Reports^®^. 2015;130(3):261–8. 10.1177/003335491513000313. 25931630 10.1177/003335491513000313 PMC4388224

[27] Appenheimer AB, Bokhour B, McInnes DK, et al. Should Human Immunodeficiency Virus Specialty clinics treat patients with hypertension or refer to primary care? An analysis of treatment outcomes. Open Forum Infect Dis. 2017;4(1):ofx005. 10.1093/ofid/ofx005. 28480278 10.1093/ofid/ofx005 PMC5413997

[28] Rosenthal JA. Qualitative Descriptors of Strength of Association and Effect size. J Soc Serv Res. 1996;21(4):37–59. 10.1300/J079v21n04_02.

[29] Reed DE, Bokhour BG, Gaj L, et al. Whole Health Use and Interest Across Veterans with Co-occurring Chronic Pain and PTSD: an examination of the 18 VA Medical Center Flagship Sites. Glob Adv Health Med. 2022;11:21649561211065374. 10.1177/21649561211065374. 35174004 10.1177/21649561211065374 PMC8841911

[30] Bokhour BG, Hyde J, Kligler B, et al. From patient outcomes to system change: evaluating the impact of VHA’s implementation of the Whole Health System of Care. Health Serv Res. 2022;57(S1):53–65. 10.1111/1475-6773.13938. 35243621 10.1111/1475-6773.13938 PMC9108226

[31] Zeliadt SB, Douglas JH, Gelman H, et al. Effectiveness of a whole health model of care emphasizing complementary and integrative health on reducing opioid use among patients with chronic pain. BMC Health Serv Res. 2022;22(1):1053. 10.1186/s12913-022-08388-2. 35978421 10.1186/s12913-022-08388-2 PMC9387037

[32] Rupcic S, Gifford AL, Fix GM. Implementing Patient-Centered Care Beyond Primary Care Settings: The Case of HIV Specialty Care. Poster presented at: Society for General Internal Medicine Annual Meeting; May 2024; Boston, MA.

[33] Green P, MacLeod CJ. SIMR: an R package for power analysis of generalized linear mixed models by simulation. Methods Ecol Evol. 2016;7(4):493–8. 10.1111/2041-210X.12504.

[34] Kumle L, Võ MLH, Draschkow D. Estimating power in (generalized) linear mixed models: an open introduction and tutorial in R. Behav Res Methods. 2021;53(6):2528–43. 10.3758/s13428-021-01546-0. 33954914 10.3758/s13428-021-01546-0 PMC8613146

[35] Palinkas LA, Horwitz SM, Green CA, Wisdom JP, Duan N, Hoagwood K. Purposeful sampling for qualitative data collection and analysis in mixed method implementation research. Adm Policy Ment Health. 2015;42(5):533–44. 10.1007/s10488-013-0528-y. 24193818 10.1007/s10488-013-0528-y PMC4012002

[36] Fix GM, Kim B, Ruben MA, McCullough MB. Direct observation methods: a practical guide for health researchers. PEC Innov. 2022;1:100036. 10.1016/j.pecinn.2022.100036. 36406296 10.1016/j.pecinn.2022.100036 PMC9670254

[37] Fix GM, VanDeusen Lukas C, Bolton RE, et al. Patient-centred care is a way of doing things: how healthcare employees conceptualize patient-centred care. Health Expect. 2018;21(1):300–7. 10.1111/hex.12615. 28841264 10.1111/hex.12615 PMC5750758

[38] Fix GM, Dryden EM, Boudreau J, Kressin NR, Gifford AL, Bokhour BG. The temporal nature of social context: insights from the daily lives of patients with HIV. PLoS ONE. 2021;16(2):e0246534. 10.1371/journal.pone.0246534. 33571283 10.1371/journal.pone.0246534 PMC7877603

[39] Fix GM, Kaitz J, Herbst AN, et al. Practical strategies for co-design: the case of engaging patients in developing patient-facing Shared-decision making materials for Lung Cancer Screening. J Patient Exp. 2024;11:23743735241252247. 10.1177/23743735241252247. 38855653 10.1177/23743735241252247 PMC11162119

[40] Glasgow RE, Vogt TM, Boles SM. Evaluating the public health impact of health promotion interventions: the RE-AIM framework. Am J Public Health. 1999;89(9):1322–7. 10.2105/ajph.89.9.1322. 10474547 10.2105/ajph.89.9.1322 PMC1508772

[41] McHugh SM, Riordan F, Curran GM, et al. Conceptual tensions and practical trade-offs in tailoring implementation interventions. Front Health Serv. 2022;2:974095. 10.3389/frhs.2022.974095. 36925816 10.3389/frhs.2022.974095 PMC10012756

[42] Dasgupta S, McManus T, Tie Y, et al. Comparison of Demographic Characteristics and Social Determinants of Health between adults with diagnosed HIV and all adults in the U.S. AJPM Focus. 2023;2(3):100115. 10.1016/j.focus.2023.100115. 37790662 10.1016/j.focus.2023.100115 PMC10546490

[43] Hyde J, Wendleton L, Fehling K, et al. Strengthening Excellence in Research through veteran Engagement (SERVE): toolkit for veteran Engagement in Research (Version 1). Veterans Health Administration, Health Services Research and Development; 2018. https://www.hsrd.research.va.gov/for_researchers/serve/.

[44] Barker A, Wiener R, Crocker D, et al. You are the Key: a co-design project to reduce disparities in black veterans’ communication with healthcare providers. Patient Exp J. 2023;10(3):27–35. 10.35680/2372-0247.1816.

